# Post-COVID Rehabilitation Outcomes: A Comparative Cohort Study

**DOI:** 10.1016/j.arrct.2025.100516

**Published:** 2025-08-21

**Authors:** Ferdinand Prüfer, Alexander Kautzky, Alexandra Unger, Špela Matko, Michael J. Fischer, Ralf Harun Zwick, Vincent Grote

**Affiliations:** a Ludwig Boltzmann Gesellschaft, Ludwig Boltzmann Institute for Rehabilitation Research, Vienna, Austria; b Department of Psychiatry and Psychotherapy, Clinical Division of Social Psychiatry, Medical University of Vienna, Vienna, Austria; c Department of Clinical Neuroscience, Division of Insurance Medicine, Karolinska Institute, Stockholm, Sweden; d University College of Teacher Education Carinthia, Viktor Frankl University College, Klagenfurt, Austria; e Institute for Outcomes Research, Center for Medical Data Science, Medical University of Vienna, Vienna, Austria; f Rehabilitation Center Kitzbühel, Kitzbühel, Austria; g Therme Wien Med, Vienna, Austria

**Keywords:** Outcome Assessment, Health Care, Physical functional performance, Postacute COVID-19 syndrome, Quality of life, Rehabilitation

## Abstract

**Objective:**

To evaluate and compare rehabilitation outcomes in patients with post-COVID syndrome (post-COVID) vs those with pulmonary, cardiovascular, metabolic, and orthopedic conditions.

**Design:**

Monocentric comparative cohort pre-post study.

**Setting:**

Outpatient rehabilitation center.

**Participants:**

Consecutive sample of 597 outpatient rehabilitation patients (N=597) (post-COVID, 227; orthopedic disorder, 147; cardiovascular disorder, 84; metabolic disorder, 83; chronic obstructive pulmonary disease [COPD], 35; asthma, 24) aged 50.3±12.7 years, 54.6% women.

**Interventions:**

Individualized, multidisciplinary outpatient rehabilitation (6-10wk, total 3.000min, minimum 3sessions/wk, and 2-3h/session) including strength/endurance training, physiotherapy, psychological support, and nutritional counseling.

**Main Outcome Measures:**

Physical function was assessed with the 6-minute walking test (6MWT), and quality of life (QOL) with the 5-level EuroQol 5-dimensional questionnaire were measured at admission and discharge of outpatient rehabilitation. Differences within and between groups were analyzed using the analysis of variance, and the effect of baseline values on the outcome’s performance was modeled.

**Results:**

The Post-COVID group was younger (44.7±12.6y), and the percentage of women (75.4%) was higher than in other outpatient rehabilitation groups. All groups improved significantly during rehabilitation. Patients with post-COVID showed the lowest baseline and discharge QOL scores. Baseline-adjusted scores demonstrated that, despite overall improvements, the post-COVID group reported significantly lower QOL than other outpatient rehabilitation groups, except the COPD group. In contrast, the post-COVID group achieved the highest physical function gains in 6MWT (+60.4m, *P*<.001). Baseline-adjusted scores indicated the highest physical function in patients with post-COVID (6MWT, PC = 632.4 m vs ALL = 603.4 m), outperforming all other outpatient rehabilitation groups. Baseline and change scores were negatively correlated, highlighting the need for baseline adjustment.

**Conclusions:**

Although outpatient rehabilitation was associated with improvements in physical function in patients with post-COVID, QOL deficits persist, discordant with other common outpatient rehabilitation indications. Targeted rehabilitation strategies addressing mental health and fatigue are needed to optimize post-COVID recovery.

Coronavirus disease 2019 (COVID-19), caused by the severe acute respiratory syndrome coronavirus 2, led to a global pandemic, infecting more than 770 million people and causing over 7 million deaths as of February 2025.^1^ Persistent symptoms after the acute infection phase, collectively known as post-COVID syndrome (PC) or long COVID, have emerged as a new global health concern.^2^, ^3^, ^4^ The mechanisms behind these persistent symptoms remain poorly understood,^4^ and therapeutic approaches based on evidence have yet to be established.

Rehabilitation is crucial for PC recovery.^5^ The World Health Organization calls for integrated, patient-centered care models for this population,^6^ including education, self-management, gradual activity resumption, breathing exercises, physical therapy, psychological support, cognitive training, and occupational therapy,^1^^,^^7^ Exercise-based rehabilitation has been advised for managing fatigue, exercise intolerance, dyspnea, mental health issues, sleep disturbances, and musculoskeletal pain.^5^^,^^7^, ^8^, ^9^, ^10^ Our previously published study^10^ found a 62.9±48.2 m increase in 6-minute walking test (6MWT) distance after a 6-week outpatient program, with 70% surpassing the minimal clinically important difference (MCID).

The effective management and prognoses of patients with PC are intricately linked to the precise evaluation of rehabilitation interventions, which is vital for gauging patient recovery.^11^, ^12^, ^13^ Such an evaluation requires both patient-reported outcome measures (PROMs) and clinician-reported outcome measures (CROMs). The 5-level EuroQol 5-dimensional questionnaire (EQ-5D-5L) as a PROM^3^^,^^14^ and the 6MWT as a CROM^15^ are well-established tools that provide key insights into patients’ well-being and physical capabilities.

Interpreting change scores in rehabilitation practice can be challenging, as absolute changes may not reflect a patient’s true rehabilitative performance. Numerically equal change scores can have different meanings depending on the baseline health status.^16^ Therefore, patients’ individual baseline values must be considered for accurate health-related quality of life (QOL) and physical performance evaluation.^17^, ^18^, ^19^ To address this, we applied the T2D performance score, an outcome metric that accounts for baseline status by integrating both discharge value and pre-post improvement,^20^, ^21^, ^22^ to enable fairer comparisons across groups (see further in the methods section).

This study compared rehabilitation outcomes of patients with PC with those of patients with pulmonary, cardiovascular, metabolic, or musculoskeletal diseases. By incorporating both conventional outcomes and baseline-adjusted T2D scores, we sought to provide a comprehensive, data-driven assessment of rehabilitation efficacy across these distinct patient cohorts undergoing outpatient rehabilitation (OPR).

## Methods

### Study design and data collection

This comparative cohort study with a pre-post design analyzed monocentric OPR data from January 2023 to December 2023 to evaluate the rehabilitation outcomes of patients with PC and compared these with those of patients with other pulmonary, cardiovascular, metabolic, or musculoskeletal diseases. Inclusion required adherence to OPR and completion of 6MWT and EQ-5D-5L at baseline (t1) and discharge (t2).

### Intervention

The OPR followed Austrian guidelines,^23^ using a multidisciplinary and interprofessional protocol including strength and endurance training, physiotherapy, psychological, and nutritional interventions.^10^ All study participants were referred by their treating physicians and underwent an individualized program lasting 6-10 weeks (3.000min total; 2.400min active therapeutic time), with therapy scheduled on at least 3 days per week. Each session lasted 2-3 hours and was distributed as evenly as possible across the week to match individual capacity and optimize recovery.

### Ethics approval

The study was conducted in accordance with the latest version of the Declaration of Helsinki and was approved and registered by the Ethics Committee of the Medical University of Vienna. All patients included in the analysis provided their written informed consent for study participation.

### Outcome measurement

We assessed age, sex, and body mass index (BMI), self-rated QOL in terms of the EQ-5D-5L index and visual analog scale (EQ-VAS), and physical performance (6MWT). Assessments were performed by trained medical staff at baseline (t1) and after outpatient rehabilitation (t2). Data were collected during routine testing and securely stored in an in-house database.

The 6MWT assesses cardiovascular and pulmonary performance below the anaerobic threshold by measuring distance walked in 6 minutes.^24^^,^^25^ Walking aids and breaks were allowed.

The QOL was measured using the well-validated EQ-5D-5L, which assesses mobility, self-care, activities, pain/discomfort, and anxiety/depression.^26^ The EQ-5D-5L index condenses all dimensions into a single value between 0 and 1, while the EQ-VAS quantifies self-rated health status on a scale ranging from 0 to 100.^27^

### Statistical analysis

SPSS^a^ (v29) was used for data analysis. Patients were grouped into (1) PC, (2) asthma, (3) chronic obstructive pulmonary disease (COPD), (4) cardiovascular, (5) metabolic, or (6) orthopedic conditions, based on their primary admission diagnosis. Score changes (Δ) from t1 to t2 were calculated. Effect sizes (Cohen’s *d*) for within-subject designs were interpreted as very small (0.01), small (0.2), medium (0.5), large (0.8), very large (1.2), and huge (2.0).^28^

Differences between and within groups were analyzed using a 6 × 2 × 2 analysis of variance (ANOVA) with the main diagnostic group (6 categories) and sex (male and female) as between-subjects factors and time (t1 and t2) as the within-subjects factor. We also tested all 2‑way interactions (group × sex, group × time, sex × time) and the 3‑way interaction (group × sex × time) in our repeated‑measures ANOVA. Effect sizes (η_p_²) were classified as 0.01 (small), 0.06 (medium), or 0.14 (large).^29^ Post hoc analysis used multiple *t*-tests (Fisher least significant difference test). We tested age and BMI as additional covariates in a preliminary analysis of covariance (ANCOVA) models. Age was highly confounded with diagnostic group, and its inclusion masked the within‑subject time effects; BMI had minimal influence on model estimates. Accordingly, only sex (which showed an independent effect on the 6MWT) was retained in the final ANOVA models.

### Performance score

Ceiling effects can occur in patients with high initial scores, limiting measurable improvement. Therefore, adjustment of an individual’s baseline values is needed. The European Medicines Agency and the US Food and Drug Administration recommend baseline adjustments in trials to enhance statistical power and precision.^30^, ^31^, ^32^, ^33^ ANCOVA and mixed-effects models address this but introduce analytical complexity, mathematical coupling, and regression-to-the-mean effects.^31^^,^^34^ The T2D performance score offers a robust alternative, allowing fair comparisons across baseline differences, while minimizing bias. The T2D performance score is a distribution-based metric that adjusts discharge scores for rehabilitation-induced improvement using the formula T2D=t2 + Δ (with Δ = changes from t1 to t2). Incorporating both final status and relative improvement, it provides a more meaningful assessment of rehabilitation outcomes.^20^, ^21^, ^22^ Unlike ANCOVA, it allows comparisons across groups with different baseline distributions in a pre-post design without mathematical coupling or regression effects.^34^ T2D scores were calculated for each patient, outcome, and group, then compared using univariate ANOVA with multiple *t*-tests for post hoc analysis. Further participants were stratified into quartiles of T2D (substantially below average, below average, above average, or substantially above average) with respect to the entire group, rather than separately for each group. This allowed us to compare group performance relative to the overall patient population. Deviations from the expected 25% within any quartile indicate whether a group performed better or worse than the overall patient population.

Scatter plots were used to show the relationship between baseline values and Δ relative to T2D scores and MCID values for each test. For the 6MWT, the MCID was set at 30 m^35^; for the EQ-5D-5L index, it was 0.063 points^36^; and for the EQ-VAS, 8 points.^37^

## Results

### Sample selection and demographic characteristics

**Fig 1:**
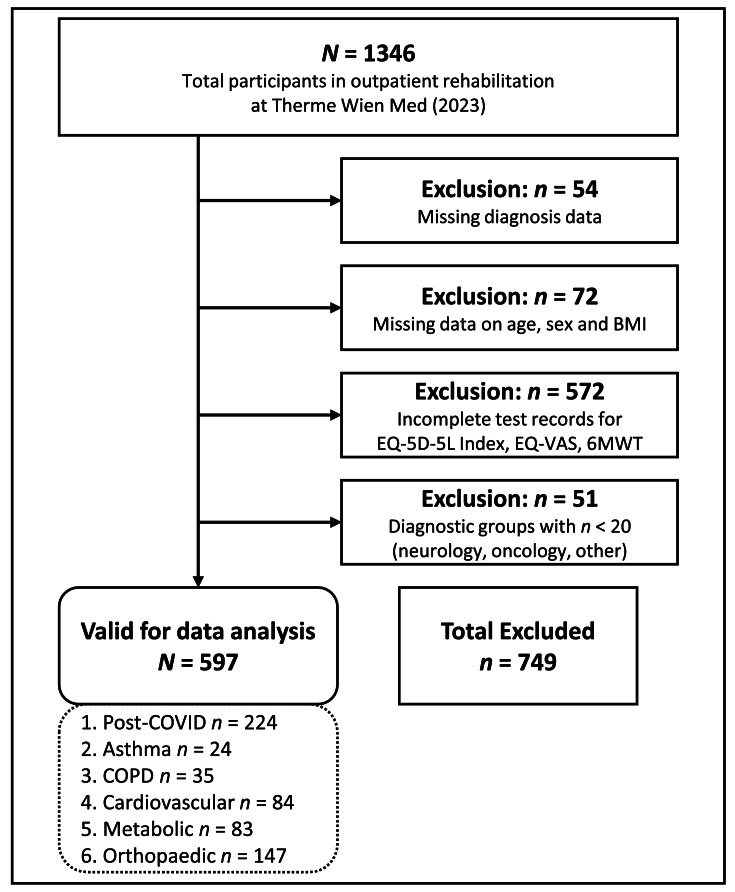
Flowchart of sample selection process.

In 2023, 1346 patients participated in OPR at the study center (Therme Wien Med). After excluding 749 patients because of missing data or small diagnostic group sizes (<20 patients), 597 remained for analysis (fig 1). Included and excluded patients did not differ significantly in age, sex, BMI, or QOL and physical performance. However, patients with PC were overrepresented, and patients with orthopedic disorders were disproportionately excluded because of missing 6MWT data (*P*<.001).

As seen in table 1, patients with PC were significantly younger than the other groups. Additionally, the PC and asthma groups had a higher proportion of women (75% and 58%, respectively), whereas the COPD, cardiovascular, and metabolic disorders groups were predominantly men (60%, 76%, and 61%, respectively; χ², *P*<.05). Mean rehabilitation duration (duration between admission and discharge) varied across groups: patients with PC averaged 70.8±54.4 days, asthma 60.6±59.7 days, COPD 53.0±30.7 days, cardiovascular disorders 62.6±46.5 days, metabolic disorders 81.0±74.7 days, and orthopedic disorders 49.1±24.4 days. The program duration for patients with metabolic disorders was significantly longer than for patients with COPD (*P*=.006), cardiovascular (*P*=.018), and orthopedic (*P*<.001) disorders. The duration of the program for patients with PC was significantly longer than for patients with an orthopedic disorder (*P*<.001).

**Table 1:** Demographics of analysis sample by diagnostic group.

Group	Age*		Sex (f/m)†
1. Post-COVID	44.7±12.6		75.4/24.6% (169/55)
2. Asthma	49.4±12.7		58.3/41.7% (14/10)
3. COPD	61.1±9.3		40.0/60.0% (14/21)
4. Cardiovascular disorder	52.5±10.6		23.8/76.2% (20/64)
5. Metabolic disorder	50.3±9.3		38.6/61.4% (32/51)
6. Orthopedic disorder	55.3±12.5		52.4/47.6% (77/70)
ALL	50.3±12.7		54.6/45.4% (326/271)

Age – Mean ± SD (y); Sex (f/m) – Sex ratio, female to male in percent and absolute.

⁎ Significant differences in univariate analysis of variance (ANOVA) for age by group, *P*<.001***; Significance of age differences between group 1 post-COVID and all other groups *P*<.001***, except group 2 asthma *p*=0.062 (*).

† Significant distribution differences in sex ratio (χ^2^ test); *** *P* ≤.001, (*) *P* ≤.1.

### Differences within time and between groups

The ANOVA (table 2) revealed significant improvements from admission to discharge across all outcomes, with 6MWT showing the largest effect (η_p_²=0.185). None of the interaction terms—group × sex, group × time, sex × time, or group × sex × time—reached statistical significance for any outcome (all *P*>.10) and are therefore not reported further. Patients with PC had significantly lower EQ-5D-5L index and EQ-VAS (see table 3) compared with the cardiovascular, metabolic, and orthopedic disorders group at both time points (*P*<.001) but achieved significantly greater 6MWT distances than those with COPD (*P*<.001) and orthopedic disorders (*P*=.016). A small interaction effect between time and group (*P*=.094; η_p_²=0.016) suggested varying degrees of improvement in walking distance among groups, with the PC group showing larger gains than COPD and asthma group (*P*<.05). Sex differences significantly affected 6MWT, as is expected for a score based on anthropometric features, with men outperforming women (PC, *P*=.005; cardiovascular, *P*<.001; and metabolic, *P*=.016 disorders), although sex had no significant effect on PROMs. Overall, patients with initial levels varied across outcome measures compared with other diagnostic groups (*P*<.001).

**Table 2:** Results of ANOVA.

	Main Effects	Interaction		Moderating factors	
ANOVA 6 × 2 × 2 N=597	Time*	Group	time × group	Sex	time × sex
	η_p_²	η_p_²	η_p_²	η_p_²	η_p_²
EQ-5D-5L index	0.016 ***	0.059 ***	0.004	0.004	0.001
EQ-VAS	0.098 ***	0.084 ***	0.012	0.000	0.001
6MWT	0.185 ***	0.063 ***	0.016 (*)	0.023 ***	0.004

η_p_² – Effect size (0.01 small, 0.06 medium, 0.14 large); 6 × 2 × 2 – 6 diagnostic groups, 2 time points, 2 sexes;

*N*=597 – total sample size; *** *P*≤.001, ** *P*≤.01, * *P*≤.05, (*) *P*≤.1.

⁎ See also table 3 for Cohen’s *d* and results of dependent *t*-test.

**Table 3:** Scores of outcome measures by diagnostic group.

Group	Admission	Discharge	Changes Δ	Cohen’s *d*	**T2D**
	EQ-5D-5L index				

1. Post-COVID	0.767±0.164	0.792±0.172	0.025±0.148	0.17 *	0.817±0.276
2. Asthma	0.815±0.167	0.830±0.152	0.015±0.141	0.11	0.845±0.241
3. COPD	0.801±0.192	0.813±0.175	0.012±0.131	0.09	0.824±0.243
4. Cardiovascular disorder	0.885±0.132	0.898±0.136	0.012±0.114	0.11	0.910±0.214
5. Metabolic dosorder	0.849±0.156	0.890±0.139	0.041±0.135	0.30 **	0.931±0.225
6. Orthopedic disorder	0.828±0.150	0.863±0.134	0.035±0.134	0.26 **	0.898±0.222
ALL	0.814±0.162	0.841±0.159	0.027±0.137	0.20 ***	0.867±0.249

	EQ-VAS				

1. Post-COVID	55.47±16.80	63.59±18.47	8.12±19.14	0.42 ***	71.71±33.66
2. Asthma	60.13±19.19	72.38±13.88	12.25±14.77	0.83 ***	84.63±21.29
3. COPD	66.37±15.57	68.34±16.42	1.97±15.23	0.13	70.31±27.59
4. Cardiovascular disorder	70.40±15.91	77.57±14.81	7.17±13.24	0.54 ***	84.74±23.15
5. Metabolic disorder	66.07±18.78	74.40±17.87	8.33±13.01	0.64 ***	82.72±24.99
6. Orthopedic disorder	63.99±17.65	70.71±18.62	6.72±19.45	0.35 ***	77.44±33.73
ALL	61.97±18.01	69.45±18.34	7.47±17.38	0.43 ***	76.92±30.86

	6MWT				

1. Post-COVID	495.4±112.9	555.8±101.4	60.4±75.4	0.80 ***	616.3±138.5
2. Asthma	529.0±142.2	559.9±99.4	30.9±76.1	0.41 (*)	590.8±105.3
3. COPD	423.6±128.7	455.4±132.4	31.8±115.2	0.28	487.2±212.2
4. Cardiovascular disorder	551.9±105.2	599.3±112.8	47.4±64.0	0.74 ***	646.7±150.3
5. Metabolic disorder	528.9±93.7	575.5±102.5	46.6±60.5	0.77 ***	622.1±139.9
6. Orthopedic disorder	484.6±115.4	531.4±121.9	46.8±54.1	0.87 ***	578.3±149.2
ALL	502.5±115.9	553.0±114.6	50.5±70.6	0.72 ***	603.4±151.0

Mean ± SD; changes Δ – discharge minus admission; significance (*P*) of differences via paired *t*-test with Cohen’s *d* as effect size; T2D – performance score T2D (discharge + difference); * *P*≤.05, ** *P*≤.01, *** *P*≤.001, (*) *P*≤.1.

### Pre-post comparisons and performance scores

**Fig 2:**
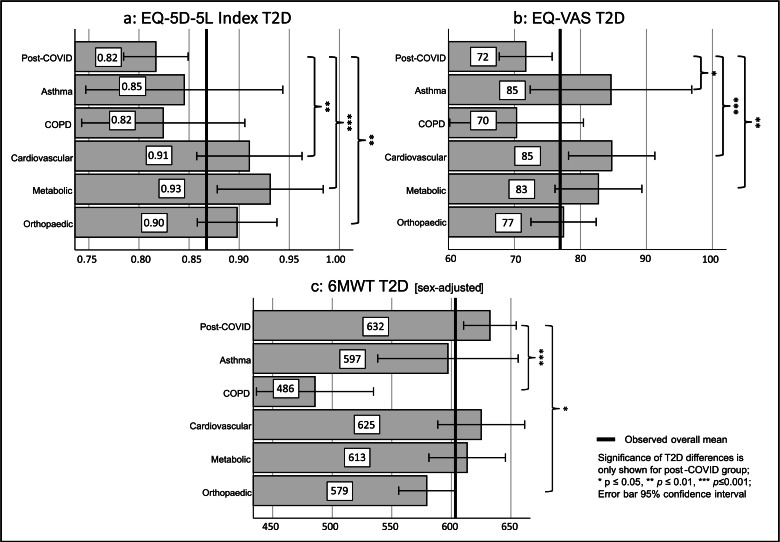
Group comparison of T2D performance scores.

Patients with PC had the lowest QOL (EQ-5D-5L index and EQ-VAS score) at both admission and discharge, whereas patients with cardiovascular disorders had the highest (table 3). Although all groups improved in the EQ-5D-5L index, significant increases were observed only in the PC (*P*=.012), metabolic (*P*=.007), and orthopedic (*P*=.002) disorder groups (Cohen’s *d*=0.17-0.30). In the EQ-VAS, all groups except COPD showed significant gains (*P*<.001; Cohen’s *d*=0.42-0.83). Patients with PC showed a higher EQ-5D-5L index Δ than patients with asthma, COPD, or cardiovascular disorders. However, EQ-5D-5L index T2D scores were significantly lower for patients with PC than those with cardiovascular (*P*=.003), metabolic (*P*<.001), or orthopedic (*P*=.002) disorders, with the PC or COPD group having the lowest T2D scores (see table 3 and fig 2A). Similarly, patients with PC experienced higher EQ-VAS Δ than those with COPD, cardiovascular, or orthopedic disorders, but significantly lower EQ-VAS T2D scores than those with asthma (*P*=.049), cardiovascular (*P*<.001), or metabolic disorders (*P*=.005) (shown in fig 2B). Patients with cardiovascular disorders reported the highest EQ-VAS T2D score, closely followed by patients with asthma, whereas patients with PC and COPD had the lowest.

For the 6MWT, patients with COPD had the lowest scores at both time points, whereas patients with cardiovascular disorders had the highest scores. All groups showed increases in walking distance, with the PC, cardiovascular, metabolic, and orthopedic disorder groups demonstrating highly significant (*P*<.001) improvements with medium to large effects (Cohen’s *d*=0.74-0.87). Although patients with PC experienced the highest 6MWT Δ, their T2D score was outperformed by patients with cardiovascular and metabolic disorders, who achieved higher walking distances at both time points. When controlling for significant effects of sex (shown in table 2), 6MWT T2D was seen to increase, particularly for the PC group (616.3±138.5 m to 632.4±168.4 m) because of the higher proportion of women compared with the other groups. As shown in figure 2C, patients with PC demonstrated the highest sex-adjusted T2D scores, significantly outperforming patients with COPD (485.5±148.0 m, *P*<.001) and orthopedic disorders (579.3±145.2 m, *P*<.014).

### Stratified performance according to T2D in patients with PC

**Fig 3:**
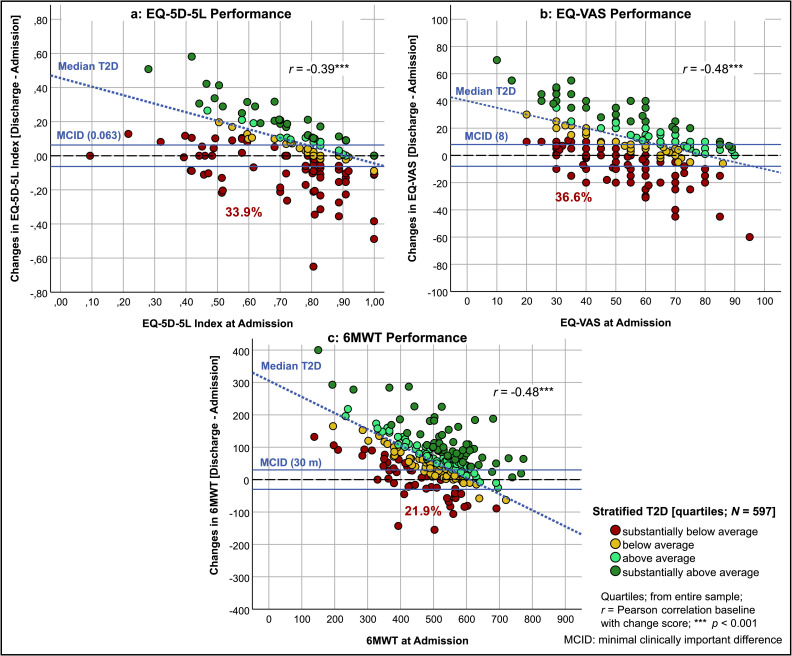
Performance of post-COVID patients stratified using T2D performance score.

Figure 3 illustrates how the performance for various PROMs and CROMs in the PC group depends on the initial values. A negative correlation was observed for the 6MWT, EQ-5D-5L index, and EQ-VAS results between the t1 and Δ scores (Pearson’s *r* ranging from −0.39 to −0.48, *P*<.001). This pattern was consistently seen for all patient groups.

As shown in table 4, more patients with PC performed substantially below average on the EQ-5D-5L index (36.6%) and EQ-VAS (33.9%) than patients in other groups, with the exception of patients with COPD regarding the EQ-VAS (37.1%). In the 6MWT, however, only 21.9% of patients with PC were assigned to the substantially below average category, outperforming patients with asthma, COPD, and orthopedic disorders, matching the metabolic disorder group (21.7%), and only being outperformed by patients with cardiovascular disorders (17.9%).

**Table 4:** Group comparison of performance categorized by T2D performance score quartiles.

Group	Measure	Substantially Below Average	Below Average	Above Average	Substantially Above Average	χ²
Post-COVID	EQ-index	33.9%	26.3%	17.9%	21.9%	.005 **
	EQ-VAS	36.6%	20.5%	24.1%	18.8%	<.001 ***
	6MWT	21.9%	25.9%	27.7%	24.6%	.658
Asthma	EQ-index	29.2%	41.7%	12.5%	16.7%	.172
	EQ-VAS	12.5%	29.2%	37.5%	20.8%	.343
	6MWT	25.0%	37.5%	12.5%	25.0%	.392
COPD	EQ-Index	31.4%	28.6%	22.9%	17.1%	.64
	EQ-VAS	37.1%	31.4%	17.1%	14.3%	.164
	6MWT	40.0%	37.1%	11.4%	11.4%	.016 *
Cardiovascular disorder	EQ-index	13.1%	29.8%	34.5%	22.6%	.033 *
	EQ-VAS	15.5%	27.4%	34.5%	22.6%	.091 (*)
	6MWT	17.9%	22.6%	22.6%	36.9%	.077 (*)
Metabolic disorder	EQ-index	14.5%	27.7%	27.7%	30.1%	.168
	EQ-VAS	18.1%	26.5%	36.1%	19.3%	.076 (*)
	6MWT	21.7%	21.7%	27.7%	28.9%	.686
Orthopedic disorder	EQ-index	21.8%	31.3%	20.4%	26.5%	.229
	EQ-VAS	25.9%	21.1%	34.0%	19.0%	.050 *
	6MWT	33.3%	21.1%	25.9%	19.7%	.084 (*)
ALL	EQ-Index	25.0%	29.0%	22.3%	23.8%	.117
	EQ-VAS	27.5%	23.5%	29.8%	19.3%	.001 ***
	6MWT	25.3%	24.8%	25.0%	25.0%	.999

Quartile classification based on T2D scores of the entire sample as a percentage; EQ-Index = EQ-5D-5L index; χ² = significance (*P*) of chi-squared test for the category distribution within groups; * *P*≤.05, ** *P*≤.01, *** *P*≤.001, (*) *P*≤.1.

Putting these results into perspective, patients with PC consistently demonstrated poorer PROMs based on the EQ-5D-5L index (T2D post-COVID, 0.82±0.28 vs T2D ALL, 0.87±0.32) and EQ-VAS (T2D post-COVID, 71.71±33.66 vs T2D ALL, 78.59±40.37) than the other diagnostic groups but showed notable improvements in the CROM (6MWT), where they achieved the highest Δ and T2D performance adjusted for sex (T2D post-COVID, 632.4±168.4 m vs T2D ALL, 588.8±199.9 m).

## Discussion

When comparing the rehabilitation outcomes of patients with PC with those of other diagnostic groups (asthma, COPD, cardiovascular, metabolic, orthopedic conditions) using the T2D performance score, the results for PROMs vs CROMs in patients with PC showed discordance.

Although significant improvements in all outcome measures (EQ-5D-5L index, EQ-VAS, and 6MWT) were observed from admission to discharge in all patient groups, patients with PC had significantly lower QOL scores than patients in other groups at both time points. Patients with PC also showed greater improvements in physical performance as assessed using the 6MWT.

The T2D results highlight this pattern: PC patients showed strong improvements in physical endurance (6MWT), but their overall rehabilitation performance, and particularly their QOL, remained low (figs 2 and 3). This finding suggests that patients with PC are physically capable of improvement, but that their subjective sense of well-being remains negatively affected, possibly reflecting the ongoing health challenges they experience even after rehabilitation.

Our findings align with those of previous research, emphasizing the role of rehabilitation in PC recovery, particularly for improving physical performance. Our previously published data^10^ show that rehabilitation can significantly improve exercise capacity and functional status in patients with PC, supporting the results of the current study for the 6MWT. Similarly, studies such as those by Chuang et al^5^ and Compagno et al^8^ highlighted the role of structured rehabilitation in improving physical outcomes, underscoring our observation that substantial improvements in walking distance occurred.

However, the lower scores obtained when applying the EQ-5D-5L index and EQ-VAS scores in PC patients suggest that these physical improvements do not necessarily translate into improved perceived health or QOL. This discrepancy has been noted in previous studies, such as that of Malik et al,^3^ where the authors documented persistent fatigue, mental health issues, and other postacute symptoms in patients with PC. The distinct effect of these ongoing symptoms on patients’ perceived QOL may explain the lower EQ-5D-5L index and EQ-VAS scores seen in our cohort. These findings are also consistent with reports that PC symptoms resemble those described for myalgic encephalomyelitis/chronic fatigue syndrome, which often includes postexertional malaise that can limit patients’ perceived recovery despite functional improvements.^38^

Our data further support the use of the T2D performance score over conventional change scores. For example, although patients with PC showed larger mean gains in PROMs compared with patients with cardiovascular disease, their absolute postrehabilitation scores remained lower (table 3). This apparent contradiction could lead to misleading interpretations if only delta values were considered. By adjusting for baseline status, the T2D score reveals that patients with cardiovascular and metabolic disorders maintained better QOL outcomes than those with PC throughout rehabilitation (fig 2). This reinforces the clinical insight that patients with lower baseline function can show greater improvements but may still not reach the absolute levels of other groups. Thus, the T2D score avoids ceiling effects and provides a more valid comparison across heterogeneous patient populations. Along these lines, we found a negative correlation between the admission and change scores for all measures, and this finding was consistent for all diagnostic groups. Notably, the MCID does not adequately capture this negative correlation in all tests. In contrast, the T2D performance score reflects this negative correlation more accurately, making it a better indicator of patient performance (fig 3).

These findings have several clinical implications. They highlighted the need for tailored rehabilitation protocols for patients with PC that address not only physical limitations but also persistent QOL issues such as fatigue and mental health concerns. We suggested the integration of mental health support, energy management strategies, and interventions to reduce postexertional malaise, in line with the recommendations of Wong and Weitzer^38^ and the WHO.^6^ In addition, the T2D performance score provides a more intuitive, personalized assessment of recovery than change scores alone, helping clinicians to track progress and identify patients who may need further support.

### Study limitations

Limitations affecting the generalisability of our results include an imbalance in the size of the diagnostic groups, particularly the overrepresentation of patients with PC compared with those with asthma and COPD. The retrospective design using routine clinical data, which, although useful for real-world applications, may introduce bias because of missing or incomplete information. Moreover, diagnostic group assignment was based solely on the primary admission diagnosis without accounting for comorbidities, which may have introduced heterogeneity within groups. Although program duration (duration between admission and discharge) varied between groups, all patients received the same prescribed total of 3,000 minutes of therapy over the course of outpatient rehabilitation, minimizing discrepancies in treatment exposure.

We did not adjust for age or BMI because of strong collinearity between age and diagnostic group and negligible effects of BMI on outcomes. Age, in particular, was unequally distributed across groups and highly confounded with diagnosis (eg, patients with PC were substantially younger than those with orthopedic or COPD diagnoses). Including age as a covariate removed meaningful time effects, despite clear within-group improvements, leading us to exclude it from the model. Future studies should investigate rehabilitation effects in age- or BMI-stratified cohorts to better understand potential interactions.

Although the T2D formula has been validated in previous studies,^19^^,^^21^ further testing in diverse populations is needed to confirm its broader applicability. Future research should explore personalized rehabilitation protocols for patients with PC, in particular, how tailored interventions might better address both physical and psychological recovery, particularly in patients experiencing postexertional malaise or fatigue.^38^ In addition, application of the T2D performance score in a wider range of rehabilitation contexts to validate its usefulness in assessing patient recovery across diagnostic groups and settings may allow researchers to overcome limitations associated with the use of traditional change scores or ANCOVA,^34^ confirming its potential as a valuable tool for assessing rehabilitation outcomes.

## Conclusions

This study clearly illustrated the complex rehabilitation trajectories of patients with PC. Despite improving in physical performance, these patients continue to experience QOL deficits. In this study, using the T2D performance score provided valuable insights into these trajectories, demonstrating its utility in assessing baseline-adjusted outcomes. The findings highlighted the need for more targeted rehabilitation interventions, particularly for addressing the long-term impacts of PC symptoms. Further research is needed to explore personalized rehabilitation strategies and the long-term effects of rehabilitation on PC recovery.

## Supplier

a. SPSS, version 29; IBM.

## Disclosure

A. K. received honoraria for presentations from Pfizer. F.P. and V.G. declared receiving following institutional funding: grant funding for the Cross-Prehab Project through Interreg Slovakia-Austria 2021-2027 program line and grant funding for the AmbRemob Project through Interreg Slovakia-Austria 2014-2020 program line by European Regional Development Fund (ERDF); grant funding for the MetaRole Project on Me/CFS 2024 by Wiener Wissenschafts-, Forschungs- und Technologiefonds (WWTF) – Vienna Science, Research and Technology Fund; and Funding for Research activities at the Ludwig Boltzmann Institute that involve open innovation in science aspects through the OIS Enrichment Fund by LBG Open Innovation in Science Center; Funding of the Rehabilitation Research Hub Austria (Rehabilitation Research in Austria) which was Co-hosted by our Ludwig Boltzmann Institute. Funding received through the Science and Innovation Strategy of the state of Salzburg (WISS 2025) by the state of Salzburg; funding for the project “Care of post covid-19 patients, effects of outpatient pneumological rehabilitation” by the Medical Scientific Fund of the Mayor of Vienna [Medizinisch-Wissenschaftlicher Fonds des Bürgermeisters der Bundeshauptstadt Wien]. This was the preceding project for the current study. The other authors have nothing to disclose.
